# Scalable synthesis of aligned carbon nanotubes bundles using green natural precursor: neem oil

**DOI:** 10.1186/1556-276X-6-92

**Published:** 2011-01-18

**Authors:** Rajesh Kumar, Radhey Shyam Tiwari, Onkar Nath Srivastava

**Affiliations:** 1 Nanoscience and Nanotechnology Unit. Department of Physics, Banaras Hindu University, Varanasi-221005, India

## Abstract

Practical application of aligned carbon nanotubes (ACNTs) would have to be determined by a matter of its economical and large-scale preparation. In this study, neem oil (also named Margoaa oil, extracted from the seeds of the neem--*Azadirachta indica*) was used as carbon source to fabricate the bundles of ACNTs. ACNTs have been synthesized by spray pyrolysis of neem oil and ferrocene mixture at 825°C. The major components of neem oil are hydrocarbon with less amount of oxygen, which provided the precursor species in spray pyrolysis growth of CNTs. The bundles of ACNTs have been grown directly inside the quartz tube. The as-grown ACNTs have been characterized through Raman spectroscopy, scanning and transmission electron microscopic (SEM/TEM) techniques. SEM images reveal that the bundles of ACNTs are densely packed and are of several microns in length. High-resolution TEM analysis reveals these nanotubes to be multi-walled CNTs. These multi-walled CNTs were found to have inner diameter between 15 and 30 nm. It was found that present technique gives high yield with high density of bundles of ACNTs.

## Introduction

Advanced carbonaceous materials have drawn great attention throughout the world because of their particular microstructures, unique properties, and great potential applications in many fields. Several carbon species such as methane, acetylene, benzene, xylene, toluene, etc., have been used as a carbon feedstock to synthesize CNTs [1-7]. These carbon precursors are related to fossil fuels which may not be sufficiently available in near future; so in order to develop a more competitive carbon material, it is necessary to consider developing carbonaceous materials from the natural resource. Recently, there have been reports on the use of natural precursor: camphor (C_10_H_16_O), turpentine oil (C_10_H_16_), eucalyptus oil (C_10_H_18_O) and palm oil (C_67_H_127_O_8_) for synthesis of CNTs [8-16]. More recently, different groups have used chemical vapor deposition method and prepared aligned carbon nanotubes (ACNTs) [17-23]. The ferrocene acts as in situ Fe catalyst precursor and forms nanosized Fe particles for the growth of ACNTs on Si substrates. Despite the mentioned efforts aiming at efficient synthesis of CNTs, further research is necessary to improve yield and purity of CNTs. The main components of neem oil are hydrocarbons containing low amount of oxygen which provides the precursor species in catalytic CVD growth of CNTs.

In this article, we report the synthesis of aligned CNTs bundles using neem oil as the carbon source using the spray pyrolysis technique. To the best of our knowledge, there has been no report on the use of this green bio-hydrocarbon in producing aligned CNTs bundles. Neem oil being a natural source which is renewable and cheap has the potential to be the green alternative for industrial scale production of CNTs.

## Experimental

Synthesis of ACNTs bundles was carried out using spray pyrolysis-assisted CVD method. Neem oil was used as carbon source and ferrocene [Fe (C_5_H_5_)_2_] as a source of Fe which acts as catalyst for the growth of ACNTs. The spray pyrolysis setup consisted of a nozzle (inner diameter 0.5 mm) attached to a ferrocene--neem oil (concentration 20 mg/ml) supply used for releasing the solution into a quartz tube (700 mm long and inner diameter 25 mm), which was mounted inside a reaction furnace (300 mm long) [24]. The outer part of the quartz tube was attached with water bubbler.

**Figure 1:**
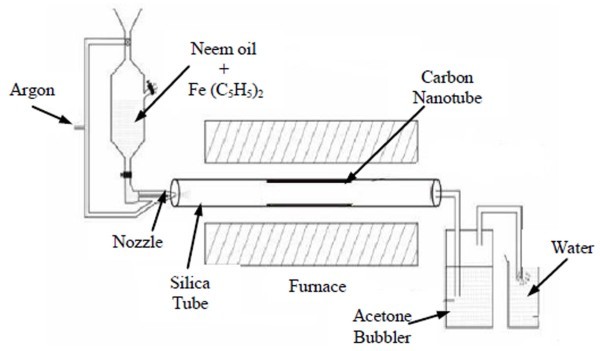
**The schematic experimental setup for the synthesis of aligned CNTs**.

In a typical experiment, the quartz tube was flushed with argon (Ar) gas first to eliminate air from the quartz tube and then heated to a reaction temperature. The precursor solution (ferrocene and neem oil) was sprayed into the quartz tube, using Ar gas. The flow rate of Ar was 80 sccm. The experiments were conducted at different temperature (750-850°C) at atmospheric pressure, with a typical reaction time of 10 min for each deposition. After deposition, the furnace was switch off and allowed to cool down to room temperature under Ar gas flow. A uniform black deposition on the inner wall of the quartz tube at the reaction hot zone was observed. The black deposition in the form of carbon soot was taken out from quartz tube. The schematic experimental setup for synthesis of aligned CNTs is given in Figure 1.

The as-grown carbon materials were characterized using scanning electron microscope (SEM) (Philips XL 20) and transmission electron microscopy (TEM) (Tecnai G^2 ^20). Raman spectroscopy was carried out with an excitation wavelength of 448 nm from Ar ion laser with typical acquisition time of 600 s. For SEM observation, the black soot-like material was directly mounted to the sample holder with silver glue which is electrically conductive. Sample for TEM studies were prepared by dispersing a small amount of black soot-like materials in ethanol with sonication for 10 min. Drops of the dispersion were placed onto a holey carbon grid and dried.

## Results and discussion

**Figure 2:**
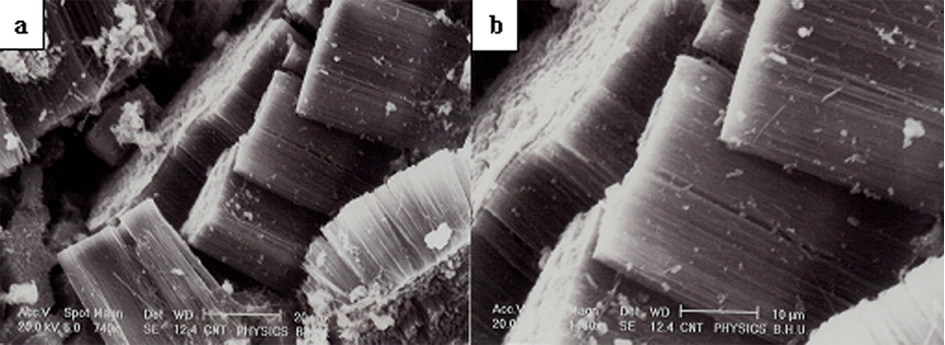
**SEM micrographs of the as-grown ACNTs at 825°C**. **(a, b) **The Cross-sectional view of the dense block of the CNTs. The complete block of ACNTs' side-view shows the parallel arrangement of the CNTs in the block.

Spray pyrolysis of ferrocene with neem oil solution at 825°C leads to large amount of carbon soot-like deposition along the total heating zone (15 cm) inside the quartz tube. Microstructural investigations of the as-grown samples were carried out using SEM and TEM techniques. The SEM study (Figure 2) reveals that the CNTs exist in the form of bundles made up of ACNTs. As can be seen, the as-grown ACNTs bundles are clean and free from other carbonaceous materials. This micrograph reveals a dense, self-aligned growth of CNTs bundles. The dense-ordered packing in the form of aligned CNTs occurs due to Vander walls interaction between the carbon nanotubes. The length of the CNTs bundles varies from 20 to 40 μm (Figure 2a). The magnified view of CNTs bundles is shown in Figure 2b. Such growth has been found for all bundles of CNTs. We repeated the experiment at 825°C temperature several times to insure the reproducibility of the formation of ACNTs bundles.

**Figure 3:**
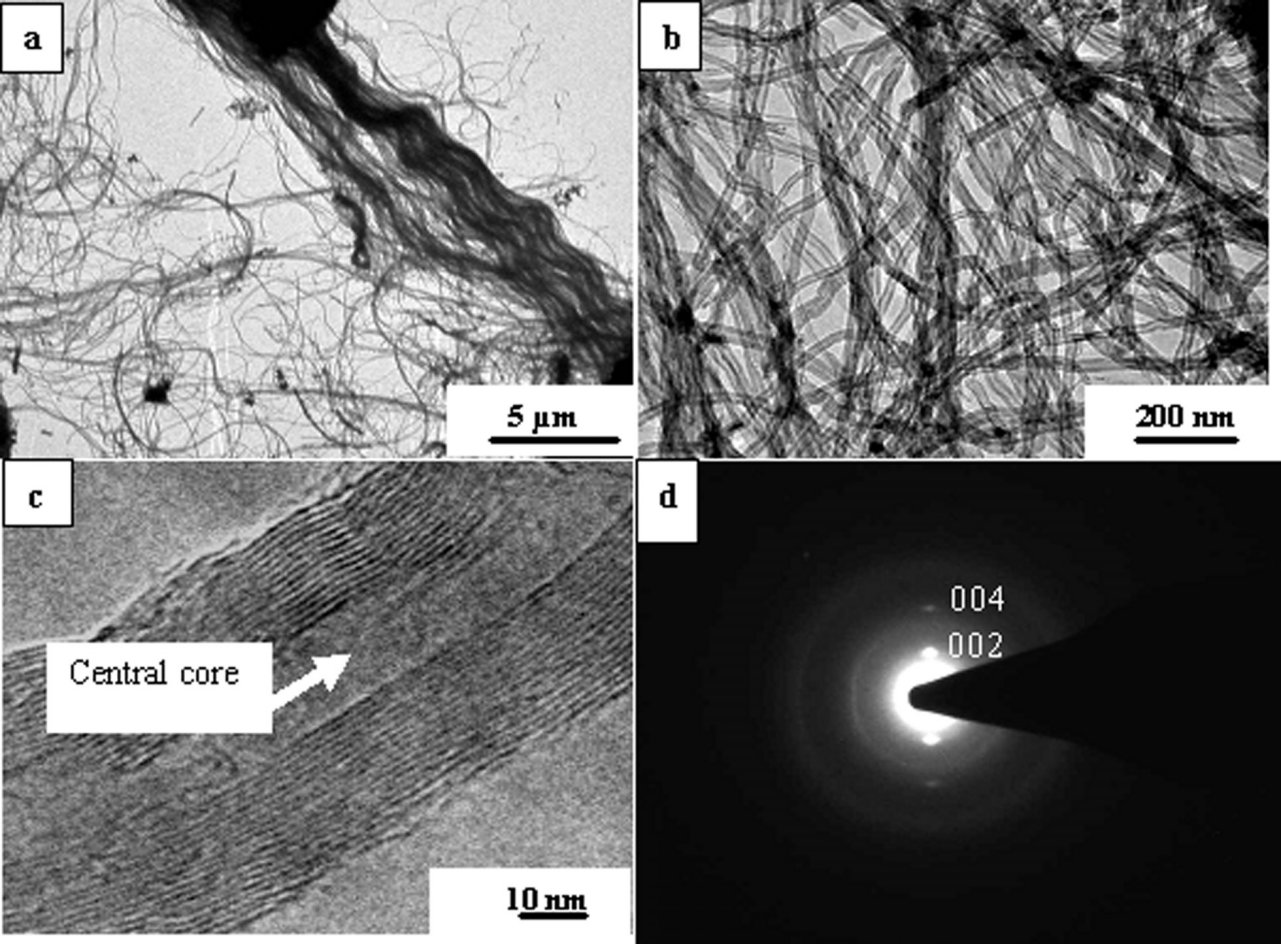
**TEM micrographs of the as-grown ACNTs at 825°C**. **(c, d) **TEM images of the as-grown CNTs. **(e) **HRTEM of CNTs and **(f) **SAED pattern.

**Figure 4:**
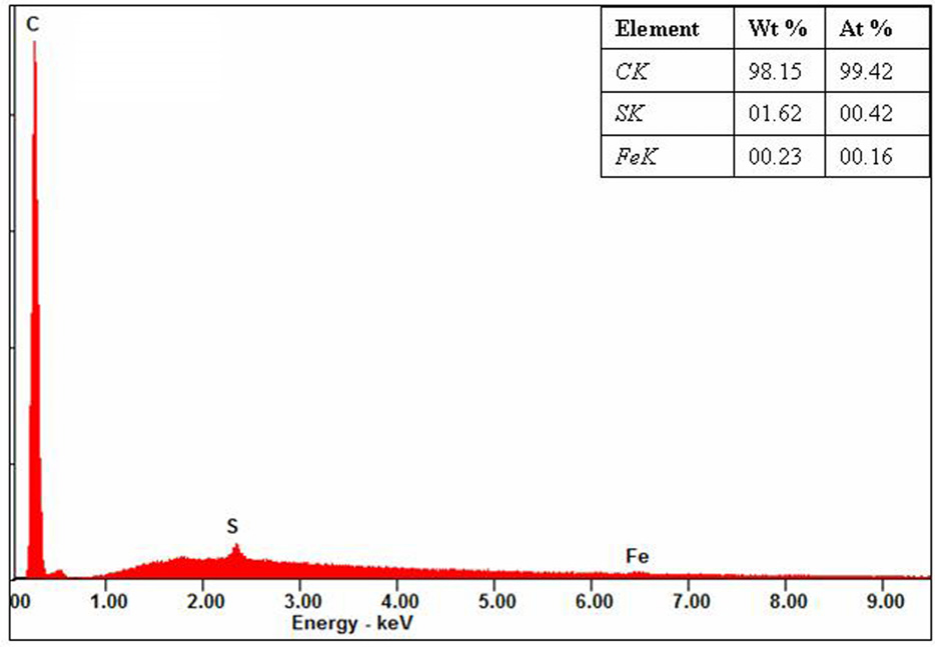
**EDX of the as-grown ACNTs at 825°C**.

Low magnification SEM images (Figure 2a, b) viewed from the lateral face and front face, respectively, depict a well-aligned array of nanotubes. Figure 3a-c shows the TEM images of CNTs grown at 825°C. It can be noticed in Figure 3b, c that amorphous carbon and metal particles are nearly absent. The magnified TEM image shows the high-density growth of CNTs having inner diameter ranging from 10 to 30 nm (Figure 3b). From TEM micrographs (Figure 3b), the approx CNT density was estimated to be of the order of 10^11 ^nanotubes/cm^2^. The high-resolution TEM (HRTEM) images of the as-grown CNTs are shown in Figure 3c. The nanotube exhibits 16 concentric graphene cylinders for which the straight fringes indicate a high degree of crystallinity. HRTEM image reveals well-graphitized MWNT layers at *d*_00.2 _lattice spacing of 0.34 nm. Energy dispersive X-ray (EDX) (Figure 4) analysis of CNTs grown at 825°C revealed the iron content of 0.23 wt%, which is in agreement with our TEM observation that metal particle in our sample is negligibly small.

**Figure 5:**
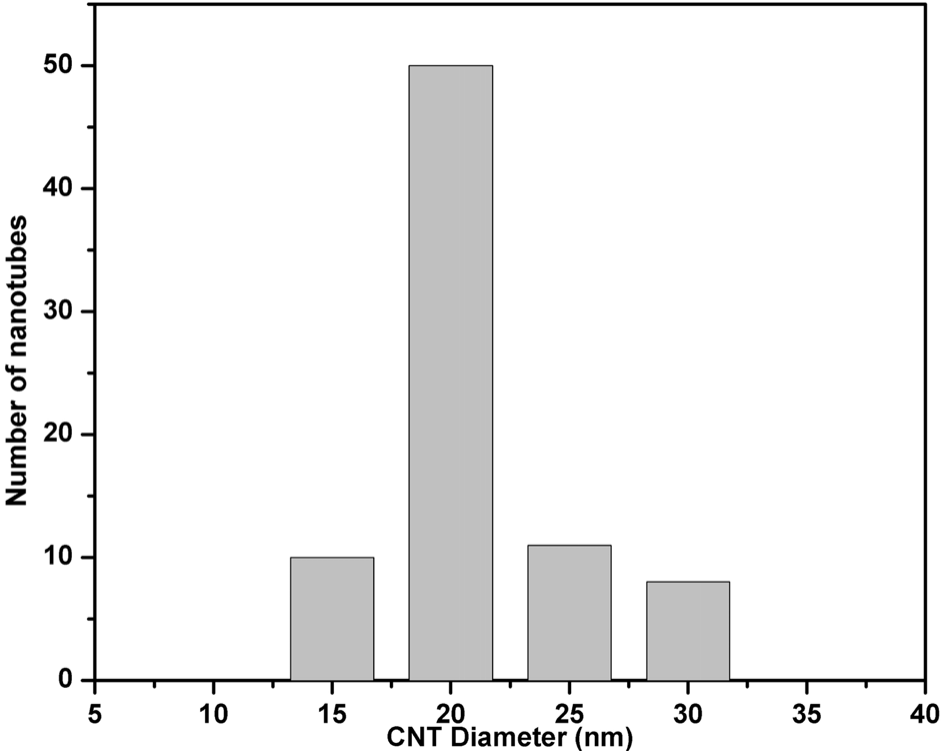
**The diameter distribution of CNTs grown at 825°C**.

The nanotubes are found to be multi-walled. These CNTs have an empty and uniform central core. A significant observation in this study is that with the use of spray pyrolysis, Fe filling in CNTs was found to be nearly absent (Figure 3c). The selected area electron diffraction (SAED) pattern taken from the CNTs shows the presence of sharp graphitic 00.2, 00.4 reflections (Figure 3d). Figure 5 shows the diameter distribution of the as-grown CNTs obtained from TEM image (Figure 3b). This shows a very narrow dispersion in the diameter of ACNT obtained in this study.

**Figure 6:**
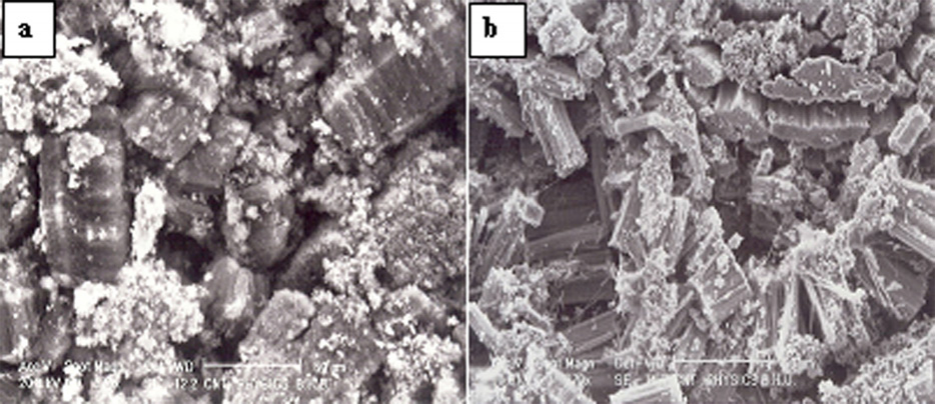
**SEM micrographs of the blocks of the ACNTs grown at (a)**. 775°C, and **(b) **875°C.

The yield of CNTs in this study was found to be determined by the growth temperature despite the fine carbon resource used to prepare CNTs. The optimum growth temperature of CNTs was 825°C. Figure 6a shows the SEM microstructure of ACNT bundles when the reaction temperature was 775°C. One can notice the poor yield of ACNT and the presence of some products due to the non-decomposition of neem oil. The formation of CNTs bundles is low because at this temperature carbon source decomposes partially leading to the formation of carbonaceous materials (e.g., amorphous carbon, etc.). On the other hand, the yield of ACNT bundles grown at 875°C was also found to be low (Figure 6b). At 875°C, the quantity of CNTs bundles within the sample has decreased (Figure 6b), and thick nanotubes have been formed. When the reaction temperature is over 875°C, some carbon black was found probably due to the high content of hydrocarbon from the decomposition of neem oil. The diameters of the ACNTs grown at 775 and 875°C were found to be 40 and 55 nm respectively. We have also used only ferrocene for synthesis of CNTs, but CNTs were not found to be aligned as well as clean.

**Figure 7:**
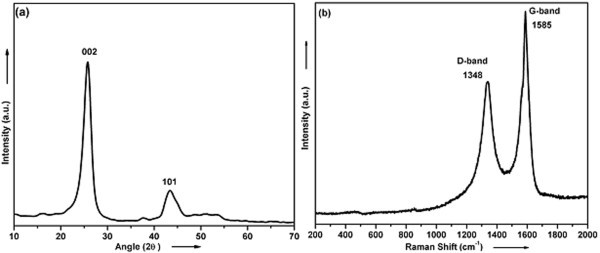
**XRD and Raman spectra of as-grown ACNTs at 825°C (a) XRD and (b) Raman spectra of the as-grown ACNTs at 825°C**.

The as-grown CNT sample was characterized by XRD. Figure 7a shows the typical XRD pattern of CNTs grown at 825°C. The peaks are indexed to be the [002] and [101] reflections of hexagonal graphite (JCPDS # 13-0148 Graphite, carbon). The presence of [002] peak in the XRD spectra of CNTs indicates that the concentric cylindrical nature of graphene sheet (*d*_002 _= 0.34 nm) nested together, and the nanotubes were multi-walled in nature. The interlayer spacing (*d*_002 _= 0.34 nm) found by XRD is consistent with that obtained (*d*_002 _~ 0.34 nm) from HRTEM (Figure 3c) and is characteristic of CNTs.

In Figure 7b, Raman-shift range 200-2000 cm^-1^, two peaks are observed at 1348 and 1585 cm^-1 ^corresponding to D and G bands, respectively. The G band corresponds to the tangential stretching (*E*_2g_) mode of the highly oriented pyrolytic graphite and suggests the CNTs to be composed of crystalline graphitic carbon. Higher intensity of G band indicates the higher degree of crystallinity/graphitization. On the other hand, the D band at 1348 cm^-1 ^originates from disorder in the *sp*^2^-hybridized carbon and indicates lattice distortions in the curved graphene sheets, tube ends, etc. The intensity ratio of D and G peaks (*I*_D_/*I*_G_) is used to characterize the degree of carbon materials, i.e., smaller ratio of *I*_D_/*I*_G _corresponds to higher degree of CNTs graphitization [25,26]. The relative intensity (*I*_D_/*I*_G_) of the as-grown CNTs is 0.265. This value reveals a higher degree of graphitization when compared to those values reported for the CNTs grown by thermal decomposition of acetylene (e.g., *I*_D_/*I*_G _~ 0.84-1.3) [27], atomic layer deposition of iron sources and oxidants (e.g. *I*_D_/*I*_G _~ 0.74-0.90) [28], and spray pyrolysis of natural precursors (*I*_D_/*I*_G _~ 0.3-0.68) [13,15,29].

**Figure 8:**
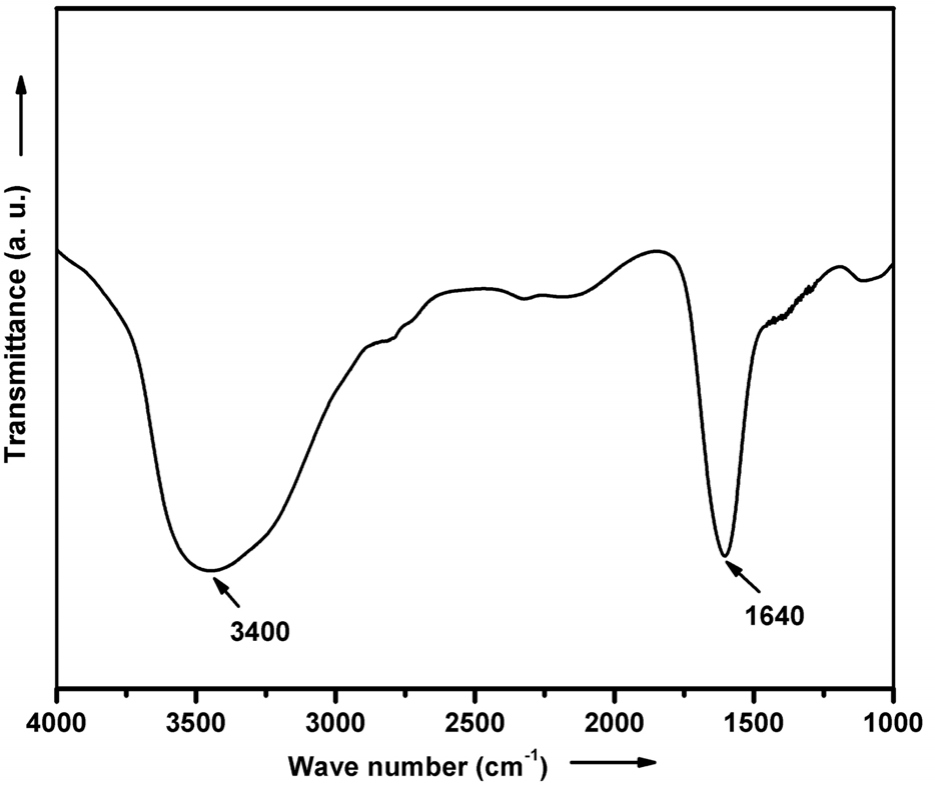
**FTIR of the as-grown ACNTs at 825°C**.

Infrared spectroscopy reveals the bonding of atoms/radicals. FTIR studies were carried out in the range of 4000-1000 cm^-1 ^to study the carbon bond in nanotubes. The FTIR spectrum of the as-grown CNTs is shown in Figure 8. The peak at 3400 cm^-1 ^is due to the presence of OH group [30], which indicates the existence of ambient atmospheric moisture in the samples. Another peak at 1640 cm^-1 ^[31] is associated with the vibration of carbon (C = C) skeleton of the CNTs. It has been suggested that no other attachments are present in place of a carbon atom.

## Conclusions

In summary, the growth of high-quality ACNTs bundles could be achieved by adjusting several processing parameters, such as ferrocene concentration in neem oil and temperature, and the same could be evidenced by TEM morphology and Raman spectroscopy. The bundles of ACNTs have been successfully prepared in large scale at 825°C under Ar atmosphere. Dense ACNT bundles with length in the range of of 20-50 μm have been formed directly inside the quartz tube. The as-grown well-crystallized multi-wall CNTs have an outer diameter in the range of 15-30 nm. The present technique gives higher yield and high density of bundles of CNTs. Graphitization of these CNTs is fairly good, and the presence of catalyst particles in the as-grown CNTs is almost negligible.

## Abbreviations

ACNTs: aligned carbon nanotubes; EDX: Energy dispersive X-ray; HRTEM: high-resolution TEM; SAED: selected area electron diffraction; SEM: scanning electron microscopy; TEM: transmission electron microscopy.

## Competing interests

The authors declare that they have no competing interests.

## Authors' contributions

RK carried out most of the experimental and drafted the manuscript. RST and ONS discussed and analyzed the experimental results.
